# Cost-effectiveness of support for health professionals to implement physical activity promotion: a protocol for within-trial and modelled economic evaluations of the PROMOTE-PA effectiveness-implementation hybrid trial

**DOI:** 10.1136/bmjopen-2024-098452

**Published:** 2025-04-05

**Authors:** Belinda Wang, Catherine Sherrington, Jennifer Cartwright, Leanne Hassett, Kate Purcell, Roslyn Savage, Anne Tiedemann, Sakina Chagpar, Daniel Cheung, Michael Noetel, Georgina Clutterbuck, Kirsten Howard, Marina Pinheiro

**Affiliations:** 1 School of Public Health, Faculty of Medicine and Health, The University of Sydney, Sydney, New South Wales, Australia; 2 Institute for Musculoskeletal Health, Sydney Local Health District, Sydney, New South Wales, Australia; 3 School of Health Sciences, Faculty of Medicine and Health, The University of Sydney, Sydney, New South Wales, Australia; 4 Western Sydney Local Health District, Sydney, New South Wales, Australia; 5 Implementation Science Academy, Sydney Health Partners, Sydney, New South Wales, Australia; 6 School of Psychology, The University of Queensland, Brisbane, Queensland, Australia; 7 School of Health and Rehabilitation Sciences, The University of Queensland, Brisbane, Queensland, Australia; 8 The Leeder Centre for Health Policy, Economics and Data, The University of Sydney, Sydney, New South Wales, Australia

**Keywords:** Exercise, Delivery of Health Care, Integrated, HEALTH ECONOMICS

## Abstract

**Introduction:**

Physical activity has important benefits for the prevention and management of chronic diseases and healthy ageing. Health professionals have valuable opportunities to promote physical activity to a large group of people across the lifespan. Promotion of Physical Activity by Health Professionals is a hybrid type 1 effectiveness-implementation cluster randomised trial designed to evaluate the impact of physical activity promotion by health professionals (n=30 clusters) on physical activity participation in their patients (n=720). To inform the future implementation of this programme, we will be conducting a within-trial and modelled economic evaluation.

**Methods and analysis:**

We will conduct a cost-effectiveness and cost-utility analysis from the perspective of the healthcare, aged care and disability funder. The time horizon will be 6 months for the within-trial analysis and 2 years for the modelled analysis. Data on intervention costs will be collected using trial records. Data on healthcare utilisation will be collected using data linkage. Incremental cost-effectiveness ratios (ICERs) will be reported for physical activity and quality-adjusted life years outcomes. Bootstrapping will be used to explore uncertainty around the ICERs and estimate 95% CIs. Results will be presented on a cost-effectiveness plane. The probability that the intervention would be cost-effective at varying willingness-to-pay thresholds will be presented using a cost-effectiveness acceptability curve.

**Ethics and dissemination:**

Ethics approval was obtained through Sydney Local Health District (RPAH zone) Ethics Review Committee (X23-0197). The findings of this study will be disseminated through peer-reviewed journal articles and conference presentations.

**Trial registration number:**

Australian New Zealand Clinical Trials Registry: ACTRN12623000920695.

**STRENGTHS AND LIMITATIONS OF THIS STUDY**

- This protocol contributes to the current limited body of evidence on the cost-effectiveness of implementation strategies supporting health professionals to promote physical activity to their patients and follows best practice recommendations for conducting and reporting an economic evaluation alongside a trial.
- Within-trial and modelled analysis will be conducted to capture costs and health outcomes over an extended timeframe, providing evidence for the long-term cost-effectiveness of implementation support for physical activity promotion.
- The use of preference-based utility scores (from EuroQol 5-dimensions 5-level version) will facilitate decision-making when comparing programmes for funding.
- The use of data linkage for healthcare utilisation costs overcomes the limitations of self-reported measures of service utilisation and will allow for a greater understanding of costs from a range of perspectives.
- The main effectiveness outcome—physical activity participation—is a self-reported measure and may be subject to recall bias.

## Introduction

 Physical activity (PA) has a multitude of health benefits, including reducing mortality and aiding in the prevention and management of chronic diseases such as cardiovascular disease, diabetes and cancer.^1^ However, physical inactivity remains a pressing global health issue, leading to 5.3 million avoidable deaths annually.^2^ To address this issue, the evaluation and widespread implementation of suitable PA interventions are urgently needed.

International organisations such as the WHO and International Society for Physical Activity and Health have advocated for PA promotion to be integrated into healthcare settings, with health professionals holding a valuable opportunity to promote PA to a large group of people across the lifespan.^3 4^ Health professionals themselves have indicated a strong interest in supporting their patients to be more active.^5 6^ However, this is limited by a number of reported barriers to providing this support, such as limited skills in facilitating behaviour change, a lack of suitable local PA options and limited knowledge of and trust in available community-based PA programmes.^5 7^

In this context, the *Promotion of PA by Health Professionals (PROMOTE-PA)* trial is a hybrid type 1 effectiveness-implementation cluster randomised trial that has been designed to investigate the effect of PA promotion by health professionals (n=30 clusters) with support from a research team on PA levels among patients (n=720) receiving care in outpatient or community settings.^8^ In addition to determining the effectiveness of the implementation strategies, decision makers will likely also be interested in whether the intervention represents value for money when considering implementation.^9 10^

Therefore, the objective of this study is to conduct an economic evaluation of PA promotion supported by implementation strategies compared with usual care of patients, with a view to inform implementation of similar programmes in other healthcare settings in the future.

### Economic questions

Primary:

1. From the perspective of healthcare, aged care and disability care funders, what is the cost-effectiveness and cost-utility (incremental costs and health outcomes in terms of PA and quality-adjusted life years (QALYs)) of PA promotion by health professionals with support from the research team compared with usual care over 6 months?

Secondary:

1. From the perspective of the healthcare funder, what is the cost-effectiveness (incremental costs and health outcomes in terms of PA promotion by health professionals (implementation outcome)) of PA promotion by health professionals with support from the research team compared with usual care over 6 months?
2. What is the level of healthcare utilisation among patients of health professionals receiving support for PA promotion compared with usual care over 2 years?
3. From the perspective of healthcare, aged care and disability care funders, what is the cost-effectiveness and cost-utility (incremental costs and health outcomes in terms of PA and QALYs) of PA promotion by health professionals with support from the research team compared with usual care over 2 years?

## Methods

An economic evaluation of a hybrid type 1 effectiveness-implementation randomised controlled trial will be conducted. A within-trial analysis will be conducted over the 6-month duration of the trial and a modelled analysis over 2 years. Healthcare utilisation over 2 years will also be reported. The trial is described briefly below; details can be found in the trial protocol published separately.^8^

### *PROMOTE-PA *trial

#### Study population

Patient participants will be 720 individuals aged 5 years and above living in the community who are receiving outpatient care from health professionals (n=30 clusters) at one of the multiple participating local health districts, a specialty children’s health network or a private clinic in New South Wales, Australia.

Study recruitment commenced in February 2024 and is expected to finish by December 2025. Participant follow-up is expected to be completed in December 2026.

#### Patient and public involvement

This study was codesigned with clinical and health service manager investigators. The implementation strategies were developed and refined with local health professional input, via semistructured interviews and focus groups during part I of *PROMOTE-PA* as well as previous surveys, interviews and consultations with health professionals and patients conducted by our group. A *PROMOTE-PA* consumer advisory group has been established for this study. Members have provided feedback on the development of study information and consent forms, participant surveys and educational resources for patients. This feedback has been used to further develop trial information and consent forms. Further details around consumer and health professional input can be found in the published trial protocol.^8^ Health professional and patient participants will be able to indicate whether they wish to receive a report of the study in the participant consent form.

#### Intervention(s) and comparator(s)

##### Intervention

As this economic evaluation is being conducted alongside an effectiveness-implementation trial, there are two components being investigated: (1) the delivery of implementation strategies to health professionals delivering outpatient and community-based care and (2) the delivery of PA promotion by health professionals to their patients (children aged 5 years or older and adults). The implementation strategies and PA promotion intervention are outlined briefly below and are further described in the trial protocol.^8^

##### Implementation strategies

The research team will deliver PA promotion support to health professional (clinical) teams using a range of implementation strategies. The implementation strategies will be tailored to each team as part of an initial service mapping process. During service mapping, the research team, together with clinical teams and managers, will collaboratively identify factors relevant to the implementation process in their clinical context and the most suitable implementation strategies to be delivered to each team. The frequency of support provided by the research team to clinical teams will vary according to the needs of each team. All teams will receive access to an online education and training resources hub, with additional implementation strategies described below:

- Community PA referral support: this may include assistance to find suitable PA opportunities and develop referral systems as well as contact with community PA opportunities from the research team.
- Experts and clinical mentors: individuals external to the clinical team sharing their experience on PA promotion (eg, through presentations and questions and answers).
- Clinical champions: individuals who are part of the clinical teams supporting implementation of PA promotion as part of routine practice.

##### PA promotion

Health professional teams will be asked to deliver PA promotion during at least one session to each patient participant as part of routine clinical practice using the 5As promotion model.^11^ All health professional teams will be advised to undertake the first component (assess their patients), with the remaining four components adopted depending on each clinical team’s implementation strategy. The 5As model includes the following components: (1) *Assess* patients’ PA levels and barriers and facilitators to participation, then provide brief PA advice; (2) *Advise* on benefits of PA and make tailored PA recommendations to their patients; (3) *Agree* on PA goals and develop a PA action plan with the patient; (4) *Assist* patients to identify physical activity barriers and potential solutions and set up a self-monitoring strategy; (5) *Arrange* patient referral to a PA programme, social support or follow-up.

Where clinical teams have limited capacity to deliver PA coaching as part of routine practice, patient participants will be invited to participate in the *PROMOTE-PA* Linkage Programme which provides up to two sessions of telehealth PA coaching to patients. Further details about this programme can be found in the trial protocol.^8^ Additional PA programmes that support patients transitioning from hospital to community-based PA programmes may be developed as required.

##### Comparator

**Figure 1:**
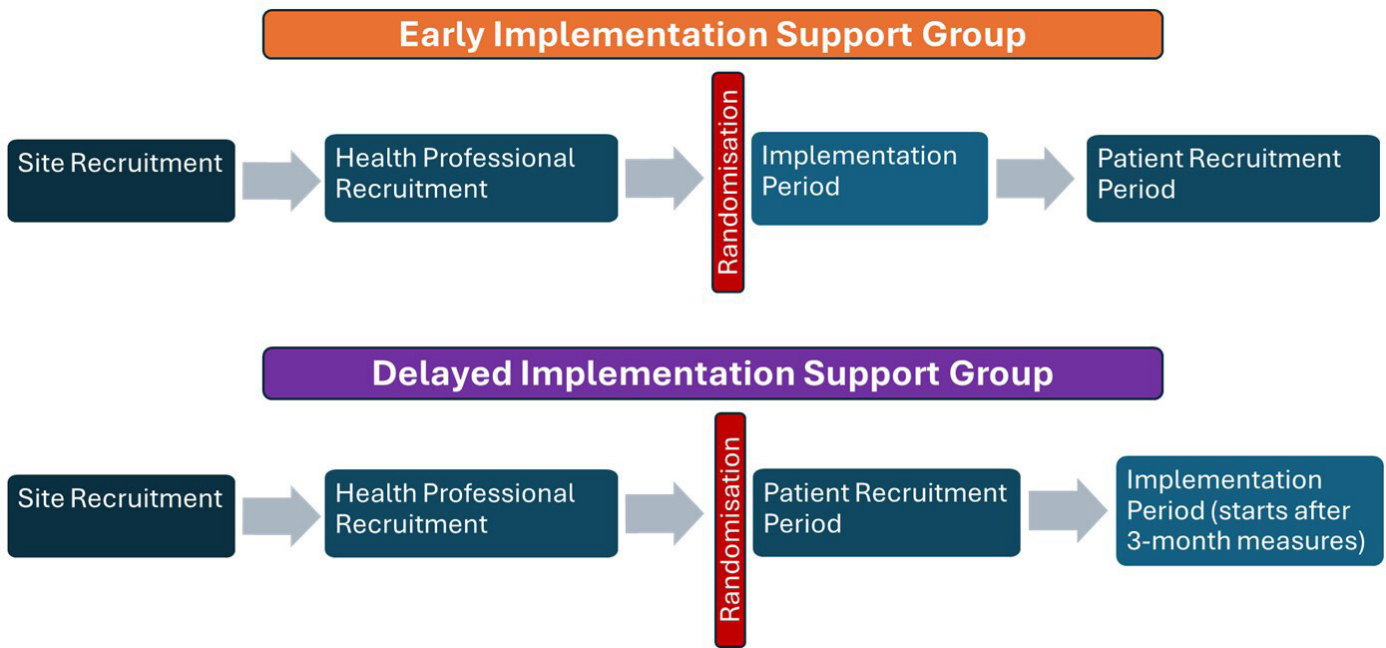
Flow of the early and delayed implementation support groups through the study.

Health professionals in the waitlist control group will not receive additional support to increase PA promotion to their patients during the control period and will continue to deliver usual care. Implementation support will be provided to these clinical teams after they have completed recruitment of their patient participants during the control period (figure 1).

### Economic evaluation overview

This economic evaluation will be conducted from the healthcare, aged care and disability care funders’ perspective. All costs will be valued in 2024 $A. Within-trial cost-effectiveness and cost-utility analysis will be conducted as part of the *PROMOTE-PA* study, to determine the incremental costs and incremental health outcomes of the implementation strategies for PA promotion compared with usual care. Data linkage will be used to determine and compare levels of healthcare utilisation among patients of health professionals receiving support for PA promotion compared with usual care over 2 years. Decision-analytical modelling will be used to determine the incremental costs and health outcomes with an extended time horizon of 2 years. The economic evaluation will be reported according to the Consolidated Health Economic Evaluation Reporting Standards.^12^ A statistical analysis plan for the within-trial and modelled analyses will be written and signed by the study authors prior to the commencement of data analysis.

### Within-trial analysis

The time horizon for the within-trial analysis is 6 months, representing the length of follow-up for patient participants in the trial. Costs and health outcomes will not be discounted as the time horizon is less than 12 months. The analysis will follow an intention-to-treat approach.

#### Measurement and valuation of health outcomes (within-trial analysis)

Intervention (patient-level) and implementation (health professional-level) outcomes will be used in this economic evaluation. The patient-level outcomes used will be the self-reported time spent in moderate-to-vigorous PA (MVPA) over the past week (reported in hours/week using a modified version of the PA vital sign)^13^ and QALYs (derived from the EuroQol 5-dimensions 5 level-version (EQ-5D-5L) or EuroQol 5-dimensions youth version (EQ-5D-Y)).^14 15^

The effectiveness outcome measure for the within-trial cost-effectiveness analysis will be the difference in proportion of participants achieving 150 min of MVPA per week in the intervention group, compared with the control group at 6 months.

The effectiveness outcome measure for the within-trial cost-utility analysis will be QALYs gained in the intervention group, compared with the control group over 6 months. Utility-based quality of life will be measured using the EQ-5D-5L^15^ in adults and the EQ-5D-Y^14^ in children and adolescents (aged 5–17 years). The EQ-5D-5L measure includes five dimensions: mobility, self-care, usual activities, pain/discomfort and anxiety/depression. Each item is rated on a five-level scale, ranging from no problems to extreme problems within each dimension. Australian scoring algorithms will be used to calculate a health utility score based on the EQ-5D-5L.^16^ The EQ-5D-Y includes the same five dimensions, reworded for relevance to children, but is instead scored on a 3-level scale. As an Australian scoring algorithm for the EQ-5D-Y has not yet been published, a valuation from a country with a similar population will be used (ie, the Netherlands).^17^ The health utility scores fall on a scale of 0.0 (dead) to 1.0 (perfect health). In the instance that a participant passes away during the trial, they will be assigned a health utility score of 0 from that point forth. Health utility scores at baseline and 3-month and 6-month follow-ups will be used to calculate QALYs using the following formula:


QALYs=healthutilityscore×timespentinhealthstate

The health professional-level outcome (secondary question) used will relate to the level of PA promoted by health professionals, captured using self-reported study-specific surveys including questions about health professionals’ PA promotion behaviours at baseline and 6-month follow-up. The effectiveness outcome measure for this within-trial cost-effectiveness analysis will be the change in PA promotion in the intervention group compared with the control group at 6 months.

#### Measurement and valuation of resource use (within-trial analysis)

Five main areas of resource use will be included in the within-trial analyses (table 1): delivery of the implementation strategy by the research team, delivery of the intervention by the health professionals, patient healthcare utilisation, use of aged care services and use of disability services. The costs of implementation and intervention delivery will be collected using trial logs, informed by the Costing implementation strategies (Cost-IS) instrument.^18^ Data to be collected on healthcare utilisation will include ambulance, hospital and community-based health service use, as well as use of medications using data linkage through the Centre for Health Record Linkage and Services Australia. Data related to the use of aged care and disability services will be collected using data linkage through Aged Care and the National Disability Insurance Scheme (NDIS). Costs will be valued using relevant state and national data.

**Table 1:** Measurement and valuation of resource utilisation

Resource items	Source of utilisation estimates	Unit of measurement	Valuation (source of unit cost)
Healthcare utilisation			
Ambulance use	Data linkage (through CHeReL)	Number of calls, cost/use	NSW ambulance fees and charges
Emergency department presentation	Data linkage (through CHeReL)	Number of emergency department presentations, (NWAU)/presentation	Urgency-related group classification system
Non-admitted patient activity	Data linkage (through CHeReL)	Number of episodes of care, NWAU/episode	Tier 2 non-admitted classification
Hospital admission	Data linkage (through CHeReL)	Number and length of admission, NWAU/admission	Australia refined diagnosis-related groups
Medicare Benefits Schedule use	Data linkage (through Services Australia)	Occasions of use, duration/occasion of use, cost/occasion of use	Medicare Benefits Schedule
Pharmaceutical Benefits Scheme use	Data linkage (through Services Australia)	Number of items, cost/item	Pharmaceutical Benefits Scheme
NDIS service use	Data linkage (through NDIS)	Occasions of use, duration/occasion of use, cost/occasion of use	NDIS pricing arrangements and price limits
Aged care service use	Data linkage (through AIHW)	Occasions of use, duration/occasion of use, cost/occasion of use	Aged care subsidies and supplements
Implementation support costs			
Staff costs			
Research staff time to conduct service mapping, planning and delivering support to health service staff (clinical teams) and updating website resources.	Trial records	Number of hours, cost/hour	University of Sydney enterprise agreement
Experts/clinical mentor time in delivering training and workshops to clinical teams.	Trial records	Number of hours, cost/hour	University of Sydney enterprise agreement/ NSW health award for the relevant year
Health service staff time participating in the service mapping process, participating in support from research staff, training/workshop activities and accessing resource and training hub.	Trial records	Number of hours, cost/hour	NSW health award for the relevant year
Travel costs	Trial records	Distance travelled, cost/km	Cents per kilometre method as per the Australia Taxation Office
Material production costs (ie, printing)	Trial records	Number of pages, cost/page	Cost as per invoice
Equipment costs (eg, activity monitors)	Trial records	Number of items, cost per item	Cost as per invoice
Website maintenance costs	Trial records	Cost/month	Cost as per invoice
Intervention delivery costs			
Health service staff time to deliver physical activity promotion.	Self-report questionnaire	Number of hours, cost/hour	NSW health award for the relevant year
Research staff time to deliver the Physical Activity Linkage Programme.	Trial records	Number of hours, cost/hour	University of Sydney enterprise agreement

AIHW, Australian Institute of Health and Welfare; CHeReL, Centre for Health Record Linkage; NDIS, National Disability Insurance Scheme; NSW, New South Wales; NWAU, National Weighted Activity Unit.

#### Analysis and sensitivity analysis (within-trial analysis)

Data analysis will be conducted using Stata V.16 (Stata Corp, College Station, Texas). The within-trial cost-effectiveness and cost-utility analyses will include total costs and effects over the 6-month trial follow-up period.

Incremental cost-effectiveness ratios (ICERs) will be calculated to determine the additional costs required to achieve the additional health outcomes associated with the implementation strategy. This will be measured in terms of gains in patient PA participation and health professional PA promotion (cost-effectiveness analysis) and QALYs (cost-utility analysis). The following formula will be used to calculate the ICER:


$$ICER=\frac{Implementation\;support\;costs\;-Usual\;care\;cost\;costs}{Implementation\;support\;effects-Usual\;care\;effects}$$

Clustering will be considered to take into account similarity within and difference between clusters when calculating the incremental costs and health outcomes.^19^ To examine uncertainty around the ICERs for PA participation, QALYs and PA promotion, bootstrapping with at least 1000 iterations will be used to estimate a distribution around costs and health outcomes, with results to be plotted on a cost-effectiveness plane. Bootstrapping will also be used to estimate 95% CIs around the ICERs. A cost-effectiveness acceptability curve will be generated to explore the probability of the intervention being cost-effective at different willingness-to-pay thresholds. To assess the robustness of results, one-way and multiway sensitivity analyses will be undertaken to see how study results change when using different assumptions and parameters (eg, under-reporting or over-reporting of self-reported PA, unit prices used for valuing resource use, methods for handling missing data).^20 21^ Cost and effectiveness outcomes will be presented as outlined in table 2.

**Table 2:** Cost and effectiveness outcomes and ICERs in base case, sensitivity and subgroup analysis

	Intervention	Usual care	Mean difference (bootstrapped 95% CI)
Base case analysis			
Total cost per participant ($A) (mean, SD)			
Total QALYs (mean, SD)			
ICER for QALYs			
Proportion of participants meeting MVPA guidelines (n, %)			
ICER for MVPA			
Scenario analysis—no extra staff cost			
Total cost per participant ($A)			
Total QALYs (mean, SD)			
ICER for QALYs			
Proportion of participants meeting MVPA guidelines (n, %)			
ICER for MVPA			
Sensitivity analysis			
Total cost per participant ($A) (mean, SD)			
Total QALYs (mean, SD)			
ICER for QALYs			
Proportion of participants meeting MVPA guidelines (n, %)			
ICER for MVPA			
Subgroup analysis			
Total cost per participant ($A) (mean, SD)			
Total QALYs (mean, SD)			
ICER for QALYs			
Proportion of participants meeting MVPA guidelines (n, %)			
ICER for MVPA			

* Intervention includes the delivery of the implementation strategy by the research team to clinical teams and the delivery of physical activity promotion by the clinical staff to their patients.

ICER, incremental cost-effectiveness ratio; MVPA, moderate-to-vigorous physical activity; QALY, quality-adjusted life year.

The base case analysis will include the costs associated with health professional time spent receiving implementation support and delivering PA promotion to account for opportunity cost. However, health professionals will be receiving implementation support during their usual working hours and delivering PA promotion as part of their routine practice. As such, a scenario analysis will be conducted where health professional time will be excluded from the total costs used to calculate the ICERs. While this approach does not account for opportunity cost, consultation with health managers indicates that such an analysis would be useful to inform the implementation of this programme.

Scenario analyses will be conducted to determine the effect of adopting different perspectives on cost-effectiveness and cost-utility. The perspectives of state and federal government funders will be considered. Exploratory subgroup analysis will be conducted in line with the analyses planned for the effectiveness outcomes (ie, subgroup analysis for paediatric participants aged 5–17 years to investigate the effect of PA promotion on PA among children and young people)^8^ and will also include cost-effectiveness and cost-utility according to socioeconomic status (as indicated by participant postcode). Subgroup analysis will be conducted for participants according to the Index of Relative Socioeconomic Disadvantage. A cut-off point of quintile two or lower will be used to represent areas with lowest Socio-Economic Indexes for Areas scores.^22^

### Comparison of resource utilisation

The primary resource utilisation outcomes will be rates and cost of healthcare, aged care and disability service utilisation in both groups over 2 years. A breakdown of healthcare utilisation costs according to cost categories (emergency, admitted, non-admitted and ambulance data, Medicare Benefits Schedule/Pharmaceutical Benefits Scheme, NDIS and aged care services use) will also be reported.

Healthcare, residential aged care and disability resource utilisation and costs will be described using means, SD, medians and IQRs over the 2-year period. Rates of service use will be compared between groups using negative binomial regression.

### Modelled economic analysis

A modelled cost-utility analysis will be conducted over an extended time horizon of 2 years. Costs and health outcomes will be discounted at 5% per annum. The outcome measure for the modelled cost-utility analyses will be the same as that for the within-trial analyses, with outcomes extrapolated to 2 years. Previous research suggests that PA interventions may result in health outcomes up to 2 years follow-up.^23^ The modelled analysis will enable the learnings from the trial to be applied to the broader context and guide future programme scale-up.

#### Model structure

A Markov model will be used to explore the long-term cost-utility of the implementation strategy with uptake and outcome data (at 6 months) from the trial. A working group will be established to define the parameters to be used in the modelled analyses. In the model, the trial cohort will be followed over 2 years. A cycle length of 3 months will be used. The 2 year healthcare, residential aged care and disability service utilisation costs will be acquired through data linkage and supported by costs reported in the published literature.

#### Analysis and sensitivity analysis (modelled analysis)

The modelled analysis results will be presented as incremental cost per QALY gained. Subgroup analysis will be conducted as per the effectiveness outcomes. Sensitivity analysis will be conducted to determine the robustness of the model. Monte Carlo simulation and probabilistic sensitivity analyses will be used to estimate a distribution around costs and health outcomes, with results to be plotted on a cost-effectiveness plane. The Monte Carlo simulation will also be used to estimate 95% CIs around the ICERs. A cost-effectiveness acceptability curve will be generated to explore the probability of the intervention being cost-effective at different willingness-to-pay thresholds.

## Discussion

In this paper, we outline the planned economic evaluation of PA promotion by health professionals with implementation strategies. We will evaluate the cost-effectiveness and cost-utility of PA promotion by health professionals with support from the research team, as well as associated healthcare utilisation. The within-trial analysis will evaluate the cost-effectiveness and cost-utility over the 6-month duration of the trial, while the modelled analysis will evaluate cost-utility over a 2-year time horizon. This extended timeframe will allow us to capture costs and health outcomes over the timeframe in which it is expected that the implementation strategies would lead to effects on patient health outcomes. Longer-term economic evaluation outcomes may also provide greater insight for decision-makers when comparing programmes for funding.

Given finite resources, economic considerations are important to inform the uptake of implementation strategies.^24 25^ Despite this, costs and economic comparisons of implementation strategies are under-reported and have been found to use inconsistent costing approaches.^26 27^ As implementation costs are likely to be borne by healthcare providers and decision-makers, the detailed capture of these costs is required to inform resource allocation within their specific setting with consideration of factors such as staff capacity, workflow and patient populations.^25 28^

As part of this economic evaluation, we are investigating the cost-effectiveness of the implementation strategies at the health professional level as well as the patient level. The economic implications of the implementation strategies at the implementation outcome level may provide valuable insights for the use of the implementation strategies in future research and practice. For example, if more resource-intensive implementation strategies are associated with limited effectiveness in changing PA promotion behaviours, this may inform decisions to adapt or prioritise alternative implementation strategies to optimise cost-effectiveness. Access to this information is likely to contribute to greater translation of evidence-based implementation strategies into practice.^28^

We acknowledge that there are some limitations to our planned economic evaluation. Given the highly tailored nature of the implementation support provided to clinical teams in the *PROMOTE-PA* trial, it is likely that the level of support, and therefore associated costs, may vary significantly across teams. This may also have implications for generalisability across clinical teams in different hospitals or local health districts operating within a different clinical context.

The primary outcome used for the trial-based cost-effectiveness component of this economic analysis is a self-reported measure of PA participation. While a sufficiently valid and reliable tool will be used, there is a risk of participants overestimating PA participation, which could impact the resulting ICER.

The primary cost-effectiveness and cost-utility analyses are being conducted from the perspective of the healthcare, aged care and disability care funder. As such, information is not being collected on patient out-of-pocket costs (eg, healthcare-related costs, equipment purchased to support an increase in PA, costs associated with signing up to gyms and sports clubs) or costs related to productivity losses. Adopting a broader perspective, such as a societal perspective, would provide a more complete indication of the economic impact of the intervention. However, given limited resources and considering the likely role of these funders in future implementation, adopting the healthcare, aged care and disability care funder perspectives is the most feasible and relevant in this case.

In this pragmatic trial, it is not feasible for health professionals to be blinded to group allocation at the time of patient recruitment. This presents a risk for potential differential recruitment. We will report baseline patient characteristics, including age, medical conditions and PA levels according to group allocation to look for evidence of differential recruitment impacts and aid in the interpretation of our findings.

Through conducting a cost-effectiveness and cost-utility analysis as well as providing detailed information around implementation, intervention and healthcare utilisation costs, this economic evaluation will contribute to increasing the knowledge base around economic evaluations of supported implementation of healthcare interventions. Understanding the cost-effectiveness and cost-utility of the implementation strategies will also inform the potential for future scale-up.

## Ethics and dissemination

Ethics approval for the *PROMOTE-PA* trial was obtained through Sydney Local Health District (Royal Prince Alfred Hospital zone) Ethics Review Committee (X23-0197). Healthcare utilisation data will be held on secure servers as required by the ethics processes for obtaining healthcare, aged care and disability data. Analysed data for the economic evaluation will be held on the University of Sydney’s Research Data Store, as per ethics requirements. The findings of this study will be disseminated through peer-reviewed journal articles and conference presentations.
