# Supplementary material for: Cost-effectiveness of support for health professionals to implement physical activity promotion: a protocol for within-trial and modelled economic evaluations of the PROMOTE-PA effectiveness-implementation hybrid trial
Source: BMJ Open. 2025 Apr 5;15(4):e098452. doi: 10.1136/bmjopen-2024-098452 (PMC11973784; doi:10.1136/bmjopen-2024-098452)
Supplement: Supplementary file 1 [file bmjopen-2024-098452.reviewer_comments.pdf]

PEER REVIEW HISTORY

BMJ Open publishes all reviews undertaken for accepted manuscripts. Reviewers are asked to complete a checklist review form and are provided with free text boxes to elaborate on their assessment. These free text comments are reproduced below.

ARTICLE DETAILS

Title (Provisional)

Cost-effectiveness of support for health professionals to implement physical activity promotion: protocol for within-trial and modelled economic evaluations of the PROMOTE-PA effectiveness-implementation hybrid trial

Authors

Wang, Belinda; Sherrington, Catherine; Baldwin, Jennifer N.; Hassett, Leanne; Purcell, Kate; Savage, Roslyn; Tiedemann, Anne; Chagpar, Sakina; Cheung, Daniel; Noetel, Michael; Clutterbuck , Georgina; Howard, Kirsten; Pinheiro , Marina

---

VERSION 1 - REVIEW

**Reviewer**	**1**
**Name**	**Pattanaprateep, Oraluck**
**Affiliation**	**Mahidol University Faculty of Medicine Ramathibodi Hospital, Department of Clinical Epidemiology and Biostatistics**
**Date**	**08-Feb-2025**
**COI**	**None**

---

some point on how to deal with missing data e.g. from data linkage process should be added. the authors will do CEA and CUA but state a lot of "benefit" words.

**Reviewer**	**2**
**Name**	**Mortimer, Duncan**
**Affiliation**	**Monash University**
**Date**	**15-Feb-2025**
**COI**	**None**

Limitations: The authors acknowledge several important limitations that arise as a consequence of the trial design but this list may be incomplete:

1. The trial contrasts an active intervention against a waitlist control and recruits patients to the intervention group after implementation but before implementation to the waitlist

group. This suggests that health providers were unblinded to receipt of the implementation strategy (if not to group assignment) when recruiting patients to the study sample, and is of potential concern because the only criteria for inclusion in the patient sample were age ≥ 5 years, a willingness to receive additional support to be physically active and absence of a medical condition precluding physical activity or study participation. The authors may wish to acknowledge the potential for differential recruitment and specify methods for identifying potentially important baseline differences, and dealing with selection of patients with higher / lower capacity to benefit into treatment / control groups.

2. Timelines specified in Figure 1 of the trial protocol suggest the potential for cross-over from the control condition to the treatment condition. Providers in the waitlist control were exposed to the intervention at 3 months from baseline, with patient recruitment to the control group occurring within the initial 3 months of the study period. The implementation period for the intervention is specified as 1-3 months and patient outcomes are measured at baseline, 3 months and 6 months. This implies that patients in the control condition who are treated by a provider who commences and completes implementation of the active intervention in less than 3 months will be 'treated' at their 6 month follow-up.

Additional detail: The authors specify a planned subgroup analysis according to socioeconomic status (as indicated by participant postcode). The authors should pre-specify definition of sub-groups; reporting number of sub-groups, measures of socio-economic status, and cut-points between sub-groups.

VERSION 1 - AUTHOR RESPONSE

**Reviewer 1 comments**	**Response**
Some point on how to deal with missing data e.g. from data linkage process should be added.	The specific methods for handling missing data will depend on the nature and amount of missing data, however we have indicated that we will be guided by the principles of handling missing data as outlined in Faria et al. (2014) and Gabrio et al. (2022).
The authors will do CEA and CUA but state a lot of "benefit" words.	We have used 'benefit' to refer to the effectiveness outcomes in this trial-based economic evaluation. To avoid confusion, we have replaced the word 'benefit' with 'health outcomes' when referring to health outcomes throughout the manuscript.
**Reviewer 2 comments**	**Response**
The trial contrasts an active intervention against a waitlist control and recruits patients to the intervention group after implementation but	We acknowledge that there is a potential for differential recruitment and have included an additional

before implementation to the waitlist group. This suggests that health providers were unblinded to receipt of the implementation strategy (if not to group assignment) when recruiting patients to the study sample, and is of potential concern because the only criteria for inclusion in the patient sample were age ≥ 5 years, a willingness to receive additional support to be physically active and absence of a medical condition precluding physical activity or study participation. The authors may wish to acknowledge the potential for differential recruitment and specify methods for identifying potentially important baseline differences, and dealing with selection of patients with higher / lower capacity to benefit into treatment / control groups.	paragraph in the discussion section to address this limitation:<br>*In this pragmatic trial, it is not feasible for health professionals to be blinded to group allocation at the time of patient recruitment. This presents a risk for potential differential recruitment. We will report baseline patient characteristics, including age, medical conditions, and physical activity levels according to group allocation to look for evidence of differential recruitment impacts and aid in the interpretation of our findings*.
Timelines specified in Figure 1 of the trial protocol suggest the potential for cross-over from the control condition to the treatment condition. Providers in the waitlist control were exposed to the intervention at 3 months from baseline, with patient recruitment to the control group occurring within the initial 3 months of the study period. The implementation period for the intervention is specified as 1-3 months and patient outcomes are measured at baseline, 3 months and 6 months. This implies that patients in the control condition who are treated by a provider who commences and completes implementation of the active intervention in less than 3 months will be 'treated' at their 6 month follow-up.	We acknowledge that at 6 months, some patient participants from the waitlist control group may have been exposed to the intervention. For this reason, our chosen primary endpoint for the effectiveness outcome is 3 months. Across the included sites, we will be collecting information on the typical number of occasions of service expected – this risk of crossover may be minimised in sites with a lower number of occasions of service. Our table of characteristics will report on anticipated number of occasions of service by site to aid in interpretation of results in relation to potential crossover.<br><br>For the economic evaluation, as we are interested in the longer-term effects of receiving the intervention, we are interested in capturing the costs and effects over a 6-month and two-year period.
The authors specify a planned subgroup analysis according to socioeconomic status (as indicated by participant postcode). The authors should pre-specify definition of sub-groups; reporting number of sub-groups, measures of socio-economic status, and cut-points between sub-groups.	We have now added the suggested detail to our planned subgroup analysis, with the revised section as following:<br><br>*Exploratory subgroup analysis will be conducted in line with the analyses planned for the effectiveness outcomes (that is, subgroup analysis for*

	paediatric participants aged 5–17 years to investigate the effect of PA promotion on PA among children and young people) and will also include cost-effectiveness and cost-utility according to socioeconomic status (as indicated by participant postcode). Subgroup analysis will be conducted for participants according to the Index of Relative Socio-economic Disadvantage (IRSD). A cut-off point of quintile 2 or lower will be used to represent areas with lowest Socio-Economic Indexes for Areas (SEIFA) scores.
